# Treatment Interruptions and Telemedicine Utilization in Serious Mental Illness: Retrospective Longitudinal Claims Analysis

**DOI:** 10.2196/33092

**Published:** 2022-03-21

**Authors:** Marcy Ainslie, Mary F Brunette, Michelle Capozzoli

**Affiliations:** 1 Department of Nursing University of New Hampshire Durham, NH United States; 2 Department of Psychiatry Geisel School of Medicine at Dartmouth Lebanon, NH United States; 3 Department of Mathematics & Statistics University of New Hampshire Durham, NH United States

**Keywords:** telemedicine, mental health, serious mental illness, retention, mental illness, telehealth

## Abstract

**Background:**

Avoiding interruptions and dropout in outpatient care can prevent mental illness symptom exacerbation and costly crisis services, such as emergency room visits and inpatient psychiatric hospitalization. During the COVID-19 pandemic, to attempt to maintain care continuity, telemedicine services were increasingly utilized, despite the lack of data on efficacy in patients with serious mental illness. Patients with serious mental illness are challenging to enroll and sustain in randomized controlled trials over time due to fluctuations in disease exacerbation. However, capturing and examining utilization and efficacy data in community mental health center (CMHC) patients with serious mental illness during the pandemic is a unique opportunity to inform future clinical and policy decision-making.

**Objective:**

We aimed to identify and describe the characteristics of CMHC patients with serious mental illness who experienced treatment interruptions and who utilized telemedicine during the pandemic.

**Methods:**

We conducted a retrospective observational study of treatment interruptions and telemedicine use during the period from December 2019 to June 2020 (compared to the period from December 2018 to June 2019) in New Hampshire CMHC patients. The study population included all Medicaid beneficiaries with serious mental illness engaged in treatment 3 months prior to the declaration of a state of emergency in response to the COVID-19 pandemic. We used chi-square tests of independence and logistic regression to explore associations between treatment interruptions and variables (gender, age, rurality, and diagnosis). Telemedicine utilization was categorized as low (<25%), medium (25%-75%), or high (>75%) use.

**Results:**

A total of 16,030 patients were identified. New Hampshire CMHCs demonstrated only a 4.9% increase in treatment interruptions compared with the year prior. Patients who were male (odds ratio [OR] 1.27, 95% CI 1.17-1.38; *P*<.001), under the age of 18 years (ages 0-12 years: OR 1.37, 95% CI 0.62-0.86, *P*<.001; aged 13-17 years: OR 1.49, 95% CI 0.57-0.79, *P*<.001), or among milder diagnostic categories, such as anxiety disorders (OR 3.77, 95% CI 3.04-4.68; *P*<.001) and posttraumatic stress disorder (OR 3.69, 95% CI 2.96-4.61; *P*<.001), were most likely to experience treatment interruptions. Patients who were female (OR 0.89, CI 0.65-0.74), 18 to 34 years old (OR 0.74, CI 0.70-0.79), or among milder diagnostic categories, such as anxiety disorder (OR 0.69, CI 0.65-0.74) or posttraumatic stress disorder (OR 0.77, CI 0.72-0.83), and with major depressive disorder (OR 0.73, CI 0.68-0.78) were less likely to be in the low telemedicine utilization group.

**Conclusions:**

The integration of telemedicine supported care continuity for most CMHC patients; yet, retention varied by subpopulation, as did telemedicine utilization. The development of policies and clinical practice guidelines requires empirical evidence on the effectiveness and limitations of telemedicine in patients with serious mental illness.

## Introduction

In response to the COVID-19 pandemic, the use of mental health telemedicine broadly expanded in US community mental health centers (CMHCs). CMHCs are designated by states to provide long-term outpatient behavioral, rehabilitation, and medication mental health services to people with serious mental illness, such as disabling schizophrenia, bipolar disorder, major depression, posttraumatic stress disorder, or other anxiety disorders [1]. More than 10 million Americans (approximately 5%) have serious mental illness, and such mental illnesses are a leading source of disability and treatment expenses [2,3], with schizophrenia alone costing approximately US $37.7 billion per year [4].

Prior to the pandemic, telemedicine was used to maintain mental health care continuity when patients and providers were separated by a distance, address transportation or childcare-related barriers, and address provider shortages [5,6]. Avoiding interruptions and dropout in outpatient care has been shown to prevent mental illness symptom exacerbation and the need for costly crisis services, such as emergency room visits and inpatient psychiatric hospitalization [7,8]. Medicaid is the most common payor for patients with serious mental illness due to related disability with resulting low income [9]. Prior research has demonstrated deficits in serious mental illness patient access, utilization, and efficacy of telemedicine as a modality for care delivery. Access concerns are related to limited digital bandwidth in rural areas and device ownership in low socioeconomic status households [10]. Some serious mental illness services require in-person contact that is not possible to deliver via telemedicine [11,12]; additionally, patients with high levels of symptoms and disorganization, which can occur with schizophrenia and bipolar disorder, may have difficulty utilizing this form of treatment.

Prior to the pandemic, CMHC telemedicine services were delivered via videoconference to a small, but growing, number of patients [13]. Typically, patients presented to the local CMHC office, where necessary electronic devices and connectivity were provided, in order to connect with a mental health provider located at a distance. In this prepandemic model, the role of the local CMHC was to mitigate telemedicine access and utilization concerns.

As the pandemic emerged in the United States, federal and state governments, followed by the Centers for Medicaid and Medicare Services, put a hold on regulatory requirements that had created barriers to telemedicine utilization in the delivery of health care services prior to the pandemic, specifically regarding Health Insurance Portability and Accountability Act–approved technology and the required location of the provider during the time of the patient visit [14]. State legislation and policy developments regarding telemedicine followed; these emergency changes broadened the scope of providers who may deliver services via telemedicine and permitted patients to receive these services from their own homes [15,16]. This transition occurred prior to addressing access and utilization concerns and despite little empiric evidence on the efficacy of such services for people with serious mental illness, in general, or for people with schizophrenia and bipolar disorders, in particular.

Research about telemedicine prior to the pandemic found that user perceptions influenced the success of its implementation, users required more technology support than was available, and reimbursement presented a barrier. Clinical efficacy trials [10,11,17-24] of mental health telemedicine utilization, albeit with small sample sizes, indicated that although addressing clinician concerns, logistical problems, technology, and staffing would be necessary [17,18], telephone-based cognitive behavioral therapy for psychosis showed high therapeutic alliance [19] and treatments by phone or video were effective for major depressive disorder [20-23], posttraumatic stress disorder [10,11], and general outpatients [24]. Telemedicine via telephone facilitated low-threshold support to 120 patients with serious mental illness to promote psychotropic mediation adherence for 6 months [25]; however, no large randomized trials broadly evaluating utilization and efficacy of telemedicine in patients with serious mental illness were identified.

Initial findings during the pandemic with respect to telemedicine for people with serious mental illness are mixed but indicate that many patients are willing and able to use video- or telephone-based telemedicine from their homes [26-29]. Another study [15] on service delivery for people with all types and severities of mental illness demonstrated a widening telemedicine utilization disparity between general and minority populations that occurred in the presence of an overall increase in mental health service utilization during the pandemic and suggested that there were increased barriers to telemedicine for minority populations. Additionally, a national survey about telemedicine utilization in patients with serious mental illness demonstrated a need for improved technical support and appointment availability, while at the same time suggesting telehealth visits can promote self-care strategies and resilience [30].

The dramatic transition to mental health telemedicine that occurred during the pandemic provides an important opportunity. The pivot to telemedicine in the serious mental illness population offers a vast, natural experiment to address the literature gap resulting from the challenges of enrolling and sustaining this population in randomized trials over time due to fluctuating symptom presentation and disease severity. Objective data on utilization of telemedicine and continuity of care in CMHC patients with serious mental illness during the pandemic will inform future clinical and policy making. It is critical to recognize the diversity that exists within the serious mental illness community, mitigate biases and assumptions regarding the prospects of telemedicine in this population, and identify characteristics of specific subgroups that may fare better or worse with treatment delivered by telemedicine. The purpose of this study was to (1) describe the characteristics of patients with serious mental illness associated with disruption in services despite the telemedicine expansion during the initial 3 months after the state of emergency declaration in response to COVID-19, (2) describe the characteristics of patients with serious mental illness who were most and least likely to use telemedicine, and (3) determine the extent to which various subpopulations utilized telemedicine to receive treatment.

## Methods

### Overview

We conducted an observational retrospective study using New Hampshire Medicaid service claims in CMHCs delivering serious mental illness services. The examination compared the 3-month period after the declaration state of emergency (study retention period) to the 3-month period prior to the declaration (study base period), encompassing December 1, 2019 through June 30, 2020. Additionally, in order to assess and account for baseline variability in treatment retention in this vulnerable population, claims were examined from 1 year prior (December 1, 2018 through June 30, 2019).

### Ethics

The University of New Hampshire institutional review board reviewed the study protocol and, given that claims data did not contain identifiable protected health information, determined that this study did not require approval.

### Study Population

Service claims for New Hampshire Medicaid beneficiaries were included in the analysis if the beneficiary (1) was active or eligible in Medicaid for at least one day within the study base or retention periods and (2) received at least one treatment service from a CMHC during the first 3 months of the study period (December 1, 2019 through February 29, 2020). Patients were excluded if they did not have a treatment service in the study base period.

### Study Periods

Due to the COVID-19 pandemic, all 10 of the New Hampshire CMHCs rapidly transitioned most services to telemedicine with patients and providers in their home environment. Clinical providers, in collaboration with patients, determined the treatment delivery modality (ie, onsite versus telemedicine). All New Hampshire CMHCs transitioned to providing at least 50% of services by telemedicine on or before April 1, 2020. March 2020 was a transitional month, and thus, was eliminated from the data set. Therefore, the defined periods were (1) the study base period, from December 1, 2019 through February 29, 2020; (2) the study retention period, from April 1, 2020 through June 30, 2020; (3) the time-trends comparison to study base period, from December 1, 2018 through February 28, 2019; and (4) the time-trends comparison to study retention period, from April 1, 2019 through June 30, 2019.

### Claims Acquisition and Preparation

New Hampshire Medicaid claims data, which included CMHC treatment service claims, patient diagnoses, and patient demographic information, were obtained from the New Hampshire Department of Health and Human Services Enterprise Business Intelligence data warehouse in November 2020. Claims for all services provided in CMHCs as defined by National Provider Identifier during the 4 study periods were included. Files were excluded if demographic data were incomplete.

Beneficiaries with CMHC treatment service claims were then selected. Treatment services were defined as codes for services that required therapeutic interaction between a mental health provider and patient (Multimedia Appendix 1). Files with case management and administrative codes that reflected activities independent of patient engagement were, therefore, excluded.

### Measures

#### Outcomes

Treatment interruption was defined as instances in which those who presented with at least one treatment claim during a base period had no treatment claim during the corresponding retention period. Telemedicine use was identified by service claim codes and categorized, based on percentage of total treatment services during the retention period, into low (<25%), medium (25%-75%), or high (>75%). The study population was described by gender (male or female), age groups (0-12 years old, 13-17 years old, 18-34 years old, 35-54 years old, or 55 years and older), ZIP code (urban, representing an area with a population greater than 10,000 people, or rural, representing an area with a population of 10,000 or less), and diagnosis, which was categorized hierarchically (in the following order: schizophrenia, bipolar disorder, major depression, posttraumatic stress disorder, anxiety disorders, and all other conditions), with a designation for each beneficiary in the base and retention periods independently, because most beneficiaries had multiple diagnoses attached to their claims. Thus, if an individual had diagnoses of schizophrenia and major depression, they were included in the schizophrenia diagnosis group.

#### Statistical Analyses

The study periods and time-trends periods were compared by characteristics of gender, age group, ZIP code, and diagnosis. Summary statistics were used to calculate the change in percentage probability of a serious mental illness treatment interruption from the time trends period to the study period. Each categorical variable in the study and time-trends retention periods were analyzed with chi-square tests for independence. Primary logistic regression included all variables and was used to examine patients who were not retained in services, and again, to examine the patients who were retained in services. The misclassification rate was the number of observations that are classified incorrectly given a cut-off probability of 0.5.

Telemedicine use (low, medium, or high) was analyzed using chi-square tests for independence. The odds ratio (OR) was the proportional odds (ie, the exponent of the estimates) with the low category as the comparator—the odds of going from 25% service use to 25%-75% and >75% service use categories combined. All analyses were performed using JMP software (version 15; SAS Institute).

## Results

CMHCs in New Hampshire experienced a 15.0% increase in the number of patients using treatment services from 2019 (n=13,456) to 2020 (n=15,471). In the study retention period, in the quarter after the state of emergency declaration, 18.3% (12,635/15,471) of serious mental illness beneficiaries were not retained in community mental health treatment; in the analogous period in 2019, 13.4% (11,492/13,456) of serious mental illness beneficiaries were not retained.

There was a 3.0% higher probability of service interruption in male patients versus female patients from 2019 to 2020 (Table 1), and the probability of service disruption increased from 2019 to 2020 in each age group (0-12 years old: 6.9%; 13-17 years old: 5.8%; 18-34 years old: 4.2%; 35-54 years old: 4.7%; 55 years and older: 3.3%).

The probability of service disruption from 2019 to 2020 increased from 5.6% for rural ZIP codes and 4.9% for urban ZIP codes, and the probability of service interruption from 2019 to 2020 increased 2% for patients with schizophrenia, 1% for patients with bipolar disorder, 4.6% for patients with major depression, 5.1% for patients with posttraumatic stress disorder, and 6.8% for patients with anxiety and all other disorders.

A logistic regression model was used to examine the association of categorical variables with age group 55 years and older serving as dependent variable for age group comparison and schizophrenia serving as the dependent variable for diagnosis comparison (Table 2).

**Figure 1:**
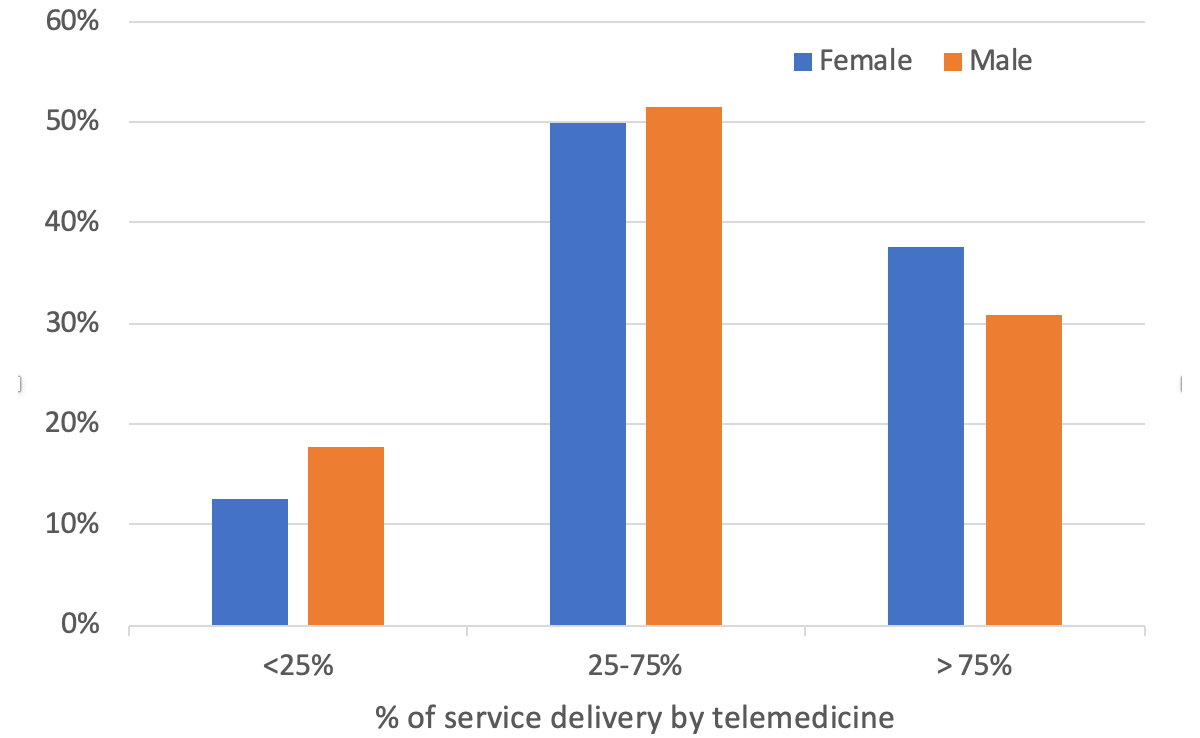
Telemedicine use by gender.

**Figure 2:**
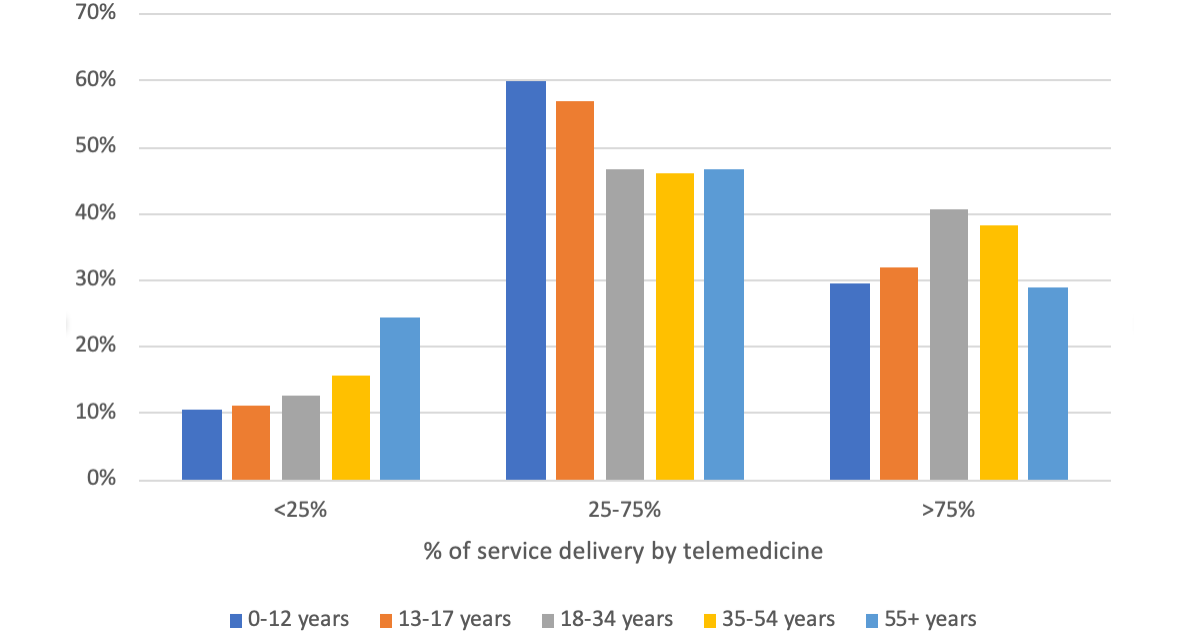
Telemedicine use by age group.

**Figure 3:**
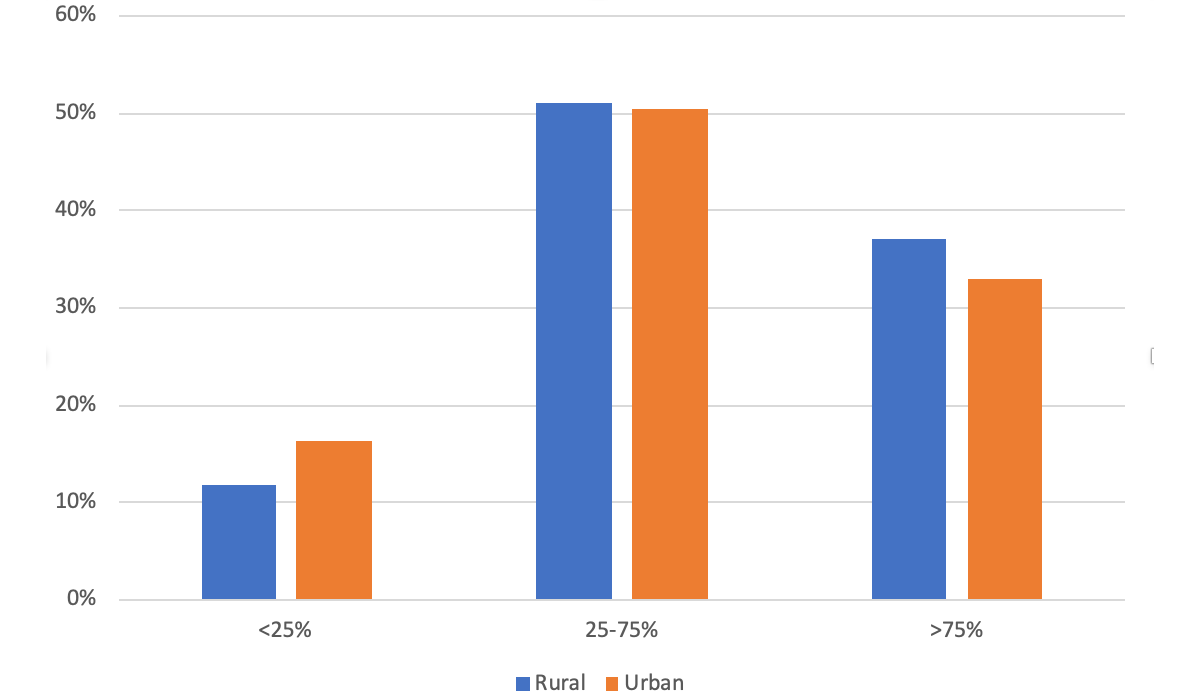
Telemedicine use by rurality.

**Figure 4:**
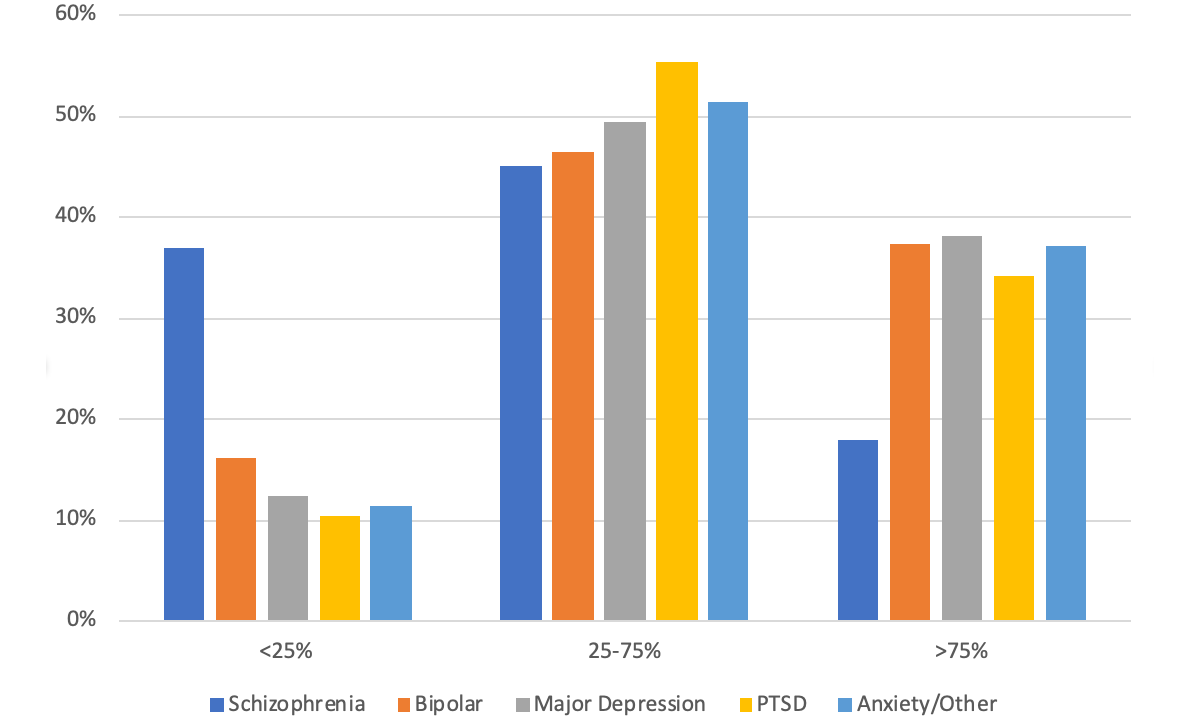
Telemedicine use by condition. PTSD: posttraumatic stress disorder.

Most beneficiaries (11,672/12427, 93.9%) participated in at least one telemedicine visit during the period from April through June 2020; in contrast, during the analogous period in 2019, a very small percentage of beneficiaries (390/13456, 2.9%) (Table 3). All subpopulations within the CMHCs were able to access and utilize telemedicine for at least a part of the treatment plan. Low, medium, and high telehealth utilization in April through June 2020 are shown by gender (Figure 1), age group (Figure 2), ZIP code (Figure 3), and diagnosis (Figure 4) categories.

Female patients had lower odds (OR 0.87, 95% CI 0.86-0.92) than male patients of going from low utilization to either moderate or high utilization. Compared with patients 55 years and older, patients 0 to 12 years old (OR 1.18, 95% CI 1.09-1.27) and 13 to 17 years old (OR 1.16, 95% CI 1.09-1.25) had greater odds and patients 18 to 34 years old (OR 0.74, 95% CI 0.70-0.79) and 35 to 54 years old (OR 0.79, 95% CI 0.74-0.84) had lower odds of going from low utilization to either moderate or high utilization. Except for patients with bipolar disorder (OR 0.93, 95% CI 0.84-1.02), patients with diagnoses other than schizophrenia had lower odds of going from low utilization to either moderate or high utilization (major depression: OR 0.73, 95% CI 0.68-0.78; posttraumatic stress disorder: OR 0.77, 95% CI 0.72-0.83; anxiety or other disorders: OR 0.69, 95% CI 0.65-0.74).

**Table 1:** CMHC patients with serious mental illness with treatment interruptions.

Characteristic		2020 (n=15,471)		2019 (n=13,456)		Change from 2019 to 2020
		Treatment interruption, n	Probability (*P*<.001), %	Treatment interruption, n	Probability (*P*=.30), %	
**Gender**						
	Female	1495	18.0	1027	14.3	3.7
	Male	1548	21.6	937	14.9	6.7
**Age group (years)**						
	0-12	689	22.3	470	15.4	6.9
	13-17	565	22.7	377	16.9	5.8
	18-34	873	23.6	551	19.4	4.2
	35-54	623	16.6	373	11.9	4.7
	≥55	293	12.1	193	8.8	3.3
**ZIP code**						
	Rural	1168	21.2	754	15.6	5.6
	Urban	1860	18.8	1191	13.9	4.9
**Diagnosis**						
	Schizophrenia	105	6.8	72	4.8	2.0
	Bipolar disorder	134	11.0	107	10.0	1.0
	Major depression	762	20.1	493	15.5	4.6
	Posttraumatic stress disorder	778	21.9	516	16.8	5.1
	Anxiety or other	1265	23.5	776	16.7	6.8

**Table 2:** Logistic regression results for patients experiencing treatment interruption.

Variable			OR^a^ (95% CI)		*P* value
**Gender**					
	Female	0.78 (0.72-0.85)		<.001	
	Male	1.27 (1.17-1.38)		<.001	
**Age (years)**					
	0-12 years	1.37 (1.17-1.61)		<.001	
	13-17 years	1.49 (1.27-1.75)		<.001	
	18-34 years	1.83 (1.58-2.12)		<.001	
	35-54 years	1.29 (1.11-1.50)		.001	
	≥55	Reference		N/A^b^	
**ZIP code**					
	Rural	1.12 (1.03-1.22)		.006	
	Urban	0.89 (0.82-0.97)		.006	
**Diagnosis**					
	Schizophrenia	Reference		N/A	
	Bipolar	1.67 (1.28-2.17)		<.001	
	Major depression	3.32 (2.67-4.13)		<.001	
	Posttraumatic stress disorder	3.69 (2.96-4.61)		<.001	
	Anxiety or other	3.77 (3.04-4.68)		<.001	

^a^OR: odds ratio.

^b^N/A: not applicable.

**Table 3:** Telemedicine utilization among patients with serious mental illness in the study retention period, after the pandemic state of emergency.

Variables				All		Low use					Medium use					High use		
			N		n		%		n			%		n			%	
**All**				12,427		1845		14.84		6294			50.64		4288			34.50
	**Gender**																	
		Female	6792		850		12.51		3394			49.97		2548			37.51	
		Male	5631		994		17.65		2897			51.45		1740			30.90	
	**Age, in years**																	
		0-12	2397		255		10.64		1436			59.91		706			29.45	
		13-17	1929		218		11.30		1096			56.82		615			31.88	
		18-34	2833		360		12.70		1321			46.63		1152			40.66	
		35-54	3132		491		15.68		1440			45.98		1201			38.34	
		55+	2132		520		24.39		998			46.81		614			28.80	
	**Diagnosis**																	
		Schizophrenia	1447		535		36.97		653			45.12		259			17.90	
		Bipolar	1080		175		16.20		502			46.48		403			37.31	
		Major depression	2949		366		12.41		1456			49.37		1127			38.21	
		Posttraumatic stress disorder	2769		291		10.51		1534			55.40		944			34.09	
		Other	4182		478		11.43		2149			51.38		1555			37.18	
	**ZIP code**																	
		Rural	4353		517		11.87		2221			51.02		1615			37.10	
		Urban	8037		1321		16.43		4056			50.46		2660			33.09	

## Discussion

Telemedicine was utilized by the majority of CMHC patients in the months following the pandemic, likely supporting continuity of care for many vulnerable patients with serious mental illness. This is consistent with the results of national surveys on telemedicine utilization in serious mental illness [27,30,31]. Yet, even with the substantial rollout of telemedicine, retention in treatment was less than retention the prior year, and some subpopulations were more at risk for treatment interruptions than others. With limited data available on telemedicine care delivery in persons with schizophrenia and bipolar disorder in particular, this data may be used to inform the decision of how best to deliver care in this population.

Older patients and patients with more severe disorders (ie, schizophrenia) were more likely to be retained in treatment; however, they were also the least likely to utilize telemedicine. This finding shows that the decision for choosing modality of care can be trusted and empowered at the clinical level, as these vulnerable patients demonstrated higher than average retention rates. The findings of this study suggest that health care professional are able to individually identify for whom and when telemedicine was a viable option. The choice of treatment modality is nuanced and may affect whether a patient is retained in treatment. With treatment retention as the overarching goal, these findings support individualized decision-making about treatment delivery modalities through patient–provider collaboration.

Female patients were more likely to use more telemedicine and more likely to be retained than male patients. Although all age groups used a lot of telemedicine services, we found that patients 55 years and older had the lowest rates of interrupted service and were in the lowest telemedicine utilization category. While some older adults require assistance navigating digital platforms [32], a systematic review [33] of telemedicine feasibility and acceptability in older adults suggested patients demonstrated high levels of feasibility and acceptability, health care providers perceived patients of this age group to have physical, sensory, cognitive, and visual–spatial challenges to successful telemedicine use. These perceptions demonstrate a bias among telemedicine use in older adult patients that is impeding this method of care delivery [33]. Based on these findings, exploring decision-making around modality choice with older adults must include recognition of individual and systemic biases that may be limiting a meaningful means of service delivery. Furthermore, adequate technical support must be put in place to ensure an equitable health care delivery system.

Youth under 18 years old, have been found, prior to the pandemic, to have high rates of acceptability and satisfaction with telemedicine services [12,34,35]; however, nevertheless, consistent with previous findings [36], the findings of our study showed that youth and adolescents had the greatest increase in service interruption compared with all other age groups year over year. Thus, the pediatric service interruptions were not expected and the cause for this should be further explored. While the under 18-year-old patient population utilized more telemedicine than most other age groups, telemedicine use during pandemic lockdowns would have required internet access, device access, and parental support or supervision. Adequate internet access was a challenge during the pandemic as work and school demands transitioned to remote access [37]. Beyond internet access, Wi-Fi–enabled devices (ie, mobile phones, laptops, and tablets) were needed by adults and children to meet this new form of engagement with work and school. Additionally, parents and caregivers were strained balancing home and work responsibilities, therefore, parents limited capabilities, to manage minor’s mental health appointments, might be expected. Finally, with most schools pivoting to web-based learning, there were fewer adults witnessing the behaviors and mental health needs of the students to encourage outreach for mental health services [38]. However, before this service interruption rate of our youth is accepted as a result of pandemic specific circumstances and not representative of broad telemedicine mental health services, root causes must be explored. Because traumatic childhood events can have a long-term impact, facilitating youth engagement in mental health services overtime must be improved.

Living in urban or rural area did not significantly impact likelihood of retention in services (*P*=.006) or telemedicine utilization (*P*=.009). In contrast, Chu et al [31] found that urban-dwelling patients demonstrated a larger increase in telemedicine utilization residing in Ontario, Canada.

Social isolation and the spread of misinformation during the pandemic has been documented to have precipitated symptom exacerbation in preexisting mental illness [39] and some psychotic events in those struggling with schizophrenia. However, stable levels of psychotic symptoms and an increase in a sense of well-being are documented in early literature exploring this disease during the COVID-19 outbreak [40]. These positive outcomes are consistent with our findings that persons with schizophrenia were the most likely beneficiaries to be retained for services during the study period. Of all diagnoses, schizophrenia demonstrated the lowest use of telemedicine services.

Offering a mixed modality of service options enabled New Hampshire CMHCs to have a very high level of retention across demographic and diagnostic variables. It should be noted in this discussion that a large majority of serious mental illness beneficiaries were able to demonstrate access, utilization, and know-how to pivot to telemedicine services for continuity of care during this exceptional public health emergency. Given the scope of challenges facing all beneficiaries and health care facilities from April to June 2020, during the onset of the pandemic, retention in services continued at a rate of only 4.9% below that of the prior year during the same time.

There were 4 main limitations to this research. First, service modality specific coding and billing modifiers used to differentiate between televideo and telephone services were not available from the outset of the state of emergency. This was likely due to parity in billing and other pressing needs during the outset of the pandemic. A national survey of patients with serious mental illness during the same period as that of our study (April through June 2020) found that approximately 64% of telemedicine visits occurred via the telephone [27] versus 23% via televideo and 13% via a combination of telephone and televideo; however, these findings were based on self-reported information and could not be validated through claims data. Second, race and ethnicity reporting in Medicaid claims was incomplete, and thus, race/ethnicity could not be included as a variable. The majority of the New Hampshire Medicaid beneficiaries receive benefits through privately managed companies for plan administration; adherence to collecting the data on race and ethnicity was poor among privately managed companies. Third, qualitative data were not collected to understand the provider–patient decision-making about choice of service modality, which would have further identified variables impacting successful engagement with care. Fourth, we collected data under the conditions of a global pandemic. It is not clear if the provider or patient behaviors and actions exhibited during this time are representative of those when there is no pandemic.

It is well documented that people with serious mental illness have greater difficulties coping with disaster events, including higher avoidance, less resilience, and a stronger likelihood of an adverse exacerbation of symptoms to a distant event, let alone one happening in the present day. Treatment continuity is critical to prevent personally and financially costly exacerbations. This research demonstrated that individualized service modality decisions to promote engagement are effectively determined between patient and provider. Furthermore, telemedicine promoted continuity of care during the pandemic across all subpopulations of CMHC patients with serious mental illness, with some populations demonstrating more utilization than others.

This research examined the initial months after the state of emergency; a more comprehensive evaluation of the effectiveness of telemedicine for people with serious mental illness is needed. Stakeholders, including patients, providers, administrators, and policymakers, require data demonstrating how best to sustain engagement in care for CMHC patients in order to make decisions about when and for whom telemedicine is efficacious.

## Appendix

Multimedia Appendix 1 Community mental health center treatment codes.

## Abbreviations

CMHC: community mental health center
OR: odds ratio
