# Supplementary material for: Treatment Interruptions and Telemedicine Utilization in Serious Mental Illness: Retrospective Longitudinal Claims Analysis
Source: JMIR Ment Health. 2022 Mar 21;9(3):e33092. doi: 10.2196/33092 (PMC8981005; doi:10.2196/33092)
Supplement: Multimedia Appendix 1 [file mental_v9i3e33092_app1.docx]

Appendix A: CMHC treatment codes

For this study, telemedicine related medical services were defined as those containing any of the following:

1) A Place of Service code of

'02'

2) A HCPCS level 1 procedure code (CPT) of

'98966', '98967', '98968',

'99421', '99422', '99423',

'99441', '99442' or '99443'

3) A HCPCS level 2 procedure code of

'G2010', 'G2012',

'G2061', 'G2062', 'G2063',

'G0406', 'G0407', 'G0408',

'G0425', 'G0426', 'G0427',

'G0459', 'G0508', 'T1014' or 'Q3014'

4) A HCPCS procedure code modifier of

'GQ', 'GT' or '95'

For reference:

[https://www.cms.gov/medicare/coding/medhcpcsgeninfo](https://nam12.safelinks.protection.outlook.com/?url=https%3A%2F%2Fwww.cms.gov%2Fmedicare%2Fcoding%2Fmedhcpcsgeninfo&data=04%7C01%7CMarcy.Ainslie%40unh.edu%7C9f3f87ef1ede4d19f4a508d90a422b25%7Cd6241893512d46dc8d2bbe47e25f5666%7C0%7C0%7C637552101564140318%7CUnknown%7CTWFpbGZsb3d8eyJWIjoiMC4wLjAwMDAiLCJQIjoiV2luMzIiLCJBTiI6Ik1haWwiLCJXVCI6Mn0%3D%7C1000&sdata=AbUGs8%2Frexd0SgSX4YzNZNY81lPbm4s8ixpzDAi7HgA%3D&reserved=0)

[https://www.cms.gov/medicare/coding/place-of-service-codes](https://nam12.safelinks.protection.outlook.com/?url=https%3A%2F%2Fwww.cms.gov%2Fmedicare%2Fcoding%2Fplace-of-service-codes&data=04%7C01%7CMarcy.Ainslie%40unh.edu%7C9f3f87ef1ede4d19f4a508d90a422b25%7Cd6241893512d46dc8d2bbe47e25f5666%7C0%7C0%7C637552101564150304%7CUnknown%7CTWFpbGZsb3d8eyJWIjoiMC4wLjAwMDAiLCJQIjoiV2luMzIiLCJBTiI6Ik1haWwiLCJXVCI6Mn0%3D%7C1000&sdata=Y3qGN%2FqWbbnc1cTbmxJ3DSxj62Ia%2Bt35C%2BiZLaloNMk%3D&reserved=0)
